# Within-Site Variation in Feather Stable Hydrogen Isotope (*δ*^2^H_f_) Values of Boreal Songbirds: Implications for Assignment to Molt Origin

**DOI:** 10.1371/journal.pone.0163957

**Published:** 2016-11-02

**Authors:** Cameron J. Nordell, Samuel Haché, Erin M. Bayne, Péter Sólymos, Kenneth R. Foster, Christine M. Godwin, Richard Krikun, Peter Pyle, Keith A. Hobson

**Affiliations:** 1 Department of Biological Sciences, University of Alberta, Edmonton, Alberta, Canada; 2 Environment and Climate Change Canada, Yellowknife, Northwest Territories, Canada; 3 Owl Moon Environmental Inc., Fort McMurray, Alberta, Canada; 4 Lesser Slave Lake Bird Observatory, Slave Lake, Alberta, Canada; 5 Institute for Bird Populations, Point Reyes Station, California, United States of America; 6 Environment and Climate Change Canada, Saskatoon, Saskatchewan, Canada; 7 Department of Biology, University of Western Ontario, London, Ontario, Canada; Centre National de la Recherche Scientifique, FRANCE

## Abstract

Understanding bird migration and dispersal is important to inform full life-cycle conservation planning. Stable hydrogen isotope ratios from feathers (*δ*^2^H_f_) can be linked to amount-weighted long-term, growing season precipitation *δ*^2^H (*δ*^2^H_p_) surfaces to create *δ*^2^H_f_ isoscapes for assignment to molt origin. However, transfer functions linking *δ*^2^H_p_ with *δ*^2^H_f_ are influenced by physiological and environmental processes. A better understanding of the causes and consequences of variation in *δ*^2^H_f_ values among individuals and species will improve the predictive ability of geographic assignment tests. We tested for effects of species, land cover, forage substrate, nest substrate, diet composition, body mass, sex, and phylogenetic relatedness on *δ*^2^H_f_ from individuals at least two years old of 21 songbird species captured during the same breeding season at a site in northeastern Alberta, Canada. For four species, we also tested for a year × species interaction effect on *δ*^2^H_f_. A model including species as single predictor received the most support (AIC weight = 0.74) in explaining variation in *δ*^2^H_f_. A species-specific variance parameter was part of all best-ranked models, suggesting variation in *δ*^2^H_f_ was not consistent among species. The second best-ranked model included a forage substrate × diet interaction term (AIC weight = 0.16). There was a significant year × species interaction effect on *δ*^2^H_f_ suggesting that interspecific differences in *δ*^2^H_f_ can differ among years. Our results suggest that within- and among-year interspecific variation in *δ*^2^H_f_ is the most important source of variance typically not being explicitly quantified in geographic assignment tests using non-specific transfer functions to convert *δ*^2^H_p_ into *δ*^2^H_f_. However, this source of variation is consistent with the range of variation from the transfer functions most commonly being propagated in assignment tests of geographic origins for passerines breeding in North America.

## Introduction

Each year, billions of birds migrate between breeding and wintering areas [1]. Migration and dispersal movements have been difficult to quantify for most of these species. Yet, this information is important to identify the spatial scale at which population dynamics are taking place and should be considered in conservation planning (e.g. [2, 3]). Recent studies have highlighted the importance of quantifying the level of migratory connectivity and extent of natal dispersal, i.e. straight-line distance moved by an individual from its natal area to its first breeding site, in full life-cycle assessments [4–6]. High migratory connectivity implies that the majority of individuals in a breeding population use the same wintering areas, whereas low migratory connectivity suggests sparsely distributed individuals from a breeding population across the species wintering grounds [7].

Intrinsic markers like naturally occurring stable isotopes of several elements can provide important information on where birds grow their feathers and have the advantage of not requiring individuals to be recaptured when assessing movement dynamics. Among stable isotopes that have been used in movement studies (i.e. C, N, H, O, and S; [8]), stable hydrogen isotope ratios (^2^H:^1^H; depicted as *δ*^2^H) are now the most commonly used marker [9]. Predictable spatial variation resulting from amount-weighted, growing-season mean precipitation (*δ*^2^H_p_) worldwide is well documented [10]. The resulting patterns of spatial variation in *δ*^2^H_p_ (i.e. isoscapes) have been used in combination with *δ*^2^H from feathers (*δ*^2^H_f_) to infer geographic origin of birds as the H in bird feathers is ultimately derived from environmental waters where the feather was grown [11, 12]. In the Nearctic-Neotropical migration system, feathers that are grown prior to fall migration (pre-basic molt; [13]) may provide information on natal or previous breeding origin in North America, whereas feathers that are grown prior to spring migration (pre-alternate molt) can be used to identify the wintering grounds of breeding individuals [14–16]. However, before assigning birds to geographic origins, *δ*^2^H_p_ values need to be adjusted to reflect the difference between local precipitation driving food webs (*δ*^2^H_p_) and *δ*^2^H_f_ (generally from regression models; hereafter “transfer functions”; [8]). Clark et al. [11] provided transfer functions for waterfowl and songbirds sampled in North America. More recently, Hobson et al. [17] found that foraging and migratory strategies were an important source of variation that influenced the transfer function applied to migratory songbirds. They suggested that *δ*^2^H_f_ differences between ground and non-ground foragers may result from differences in ground-level evapotranspiration [17] or differences in trophic level [18].

While differences between groups of species in the transfer function between *δ*^2^H_f_ and *δ*^2^H_p_ have been documented, what causes this interspecific variation is not as well understood. Analytical approaches have been developed to deal with this uncertainty in Bayesian assignment tests to provide more accurate and less biased estimates of likely geographic origin [19]. However, a better understanding of variation in *δ*^2^H_f_ among species with the same geographic origin should help in assessing those factors contributing to variation we see among various transfer functions. For example, evaporative processes in response to increased activity or ambient conditions may lead to higher *δ*^2^H in tissues [20]. Thus, birds nesting in open areas (e.g. unforested and forest canopy) could have higher *δ*^2^H_f_ values relative to species associated with forest understory. Increased body water loss due to metabolic activity can result in enriched heavy isotopes in the body water pool, in turn leading to higher *δ*^2^H_f_ values, and this effect might be more important in smaller individuals/species [21, 22]. However, Betini et al. [23] reported the opposite pattern, where body mass of nestling Tree Swallows (*Trachycineta bicolor*) was positively correlated with *δ*^2^H from blood samples. Phylogenetically conserved life history traits [24, 25] and, potentially, conserved biochemical pathways controlling H isotope discrimination in tissues [26, 27] among closely related species suggest a potentially important phylogenetic component of *δ*^2^H_f_ that could explain important interspecific variation. Inter-annual variation in *δ*^2^H_f_ has been reported for a few songbird species [28, 29] and, all things being equal, this variation should be consistent across species nesting in the same region but, to our knowledge, no studies have tested this assumption by quantifying the species × year interaction effect on variation in *δ*^2^H_f_ of forest songbirds. Finally, different species may seek out different local microhabitats during the molt period and these might also account for differences in *δ*^2^H_f_ from the same general area but little is known about habitat use during this typically “secretive” period of the annual cycle (e.g. [30]).

In this study, we used tail feathers collected from different songbird species during the same breeding season and geographic area (within a ca. 130 km radius) to test various hypotheses that have been proposed to explain intraspecific and interspecific variation in *δ*^2^H_f_. Specifically, we used a model selection approach to compare the relative importance of species, land cover type, forage substrate, nest substrate, diet composition, body mass, sex, and phylogenetic relatedness in explaining variation in *δ*^2^H_f_ among experienced breeders (after second-year; ASY) of 21 songbird species (7 families and 16 genera). We predicted that ground foragers and insectivores (i.e. species feeding almost exclusively on invertebrates during the breeding season) would have higher *δ*^2^H_f_ compared to non-ground foragers and omnivores (i.e. species feeding on seeds, fruits, and insects during the breeding season), respectively. Observed *δ*^2^H_f_ for ground and non-ground foragers were used to validate predicted values from the transfer functions provided by Hobson et al. [17]. We also predicted that individuals nesting in open areas would have higher *δ*^2^H_f_ than those nesting in forest understory. Finally, we predicted that closely related species (i.e. within the same genera) would have more similar *δ*^2^H_f_ values than less related individuals. There were no *a priori* expectations regarding the direction of the effect (positive or negative) of body mass and sex (male or female having higher *δ*^2^H_f_) on *δ*^2^H_f_. Females are more likely to experience breeding dispersal movements, i.e. straight-line distance moved by an individual from a breeding territory to another in subsequent years, and over larger distances than males [31]. Thus, we predicted that females would have similar mean *δ*^2^H_f_ values than males, but larger variation, assuming no bias in direction of dispersal movements. For four of our most common species, we also tested for a year × species interaction effect on *δ*^2^H_f_ to determine whether annual variations in *δ*^2^H_f_ are consistent among species.

## Methods

### Study area and feather samples

The study was conducted at bird banding stations in the Lesser Slave Lake Provincial Park (Lesser Slave Lake Bird Observatory, LSLBO; 55°20' N, 114°40' W) and the lower Athabasca oil sands region (Owl Moon Environmental Inc.; 56°43' N, 111°22' W) in northeastern Alberta, Canada. The region is characterized by conifer (black spruce, *Picea mariana*; white spruce, *Picea glauca*; jack pine, *Pinus banksiana*), deciduous (trembling aspen, *Populus tremuloides*), and mixedwood stands typical of the western boreal forest. The banding stations in the oil sands region are similarly forested, but also occur in riparian areas and reclaimed mine sites.

Breeding birds have been captured at banding stations as part of the Monitoring Avian Productivity and Survivorship (MAPS) program [32]. In 2013, 32 MAPS stations were monitored in the oil sands region (Table 1). Each banding station operated from 8 to 14 mist nets (each net was 12 m × 2.6 m) and captured birds passively (i.e. no attempts were made to attract the birds). Starting at sunrise, birds were captured at each station for 6 hours every 10 days throughout the breeding season (mid-June–mid-August 2013; periods 5–10; [32]). The same year, LSLBO monitored 4 MAPS stations within a forested area of approximately 3 ha that bordered the eastern shore of Lesser Slave Lake (Table 1). In 2011, active netting was conducted within ca. 1 km of the closest MAPS station where males of four species (Ovenbird; *Seiurus aurocapilla*, Swainson's Thrush; *Catharus ustulatus*, American Redstart; *Setophaga ruticilla*, and Yellow-rumped Warbler; *Setophaga coronata*) were attracted to mist nets using conspecific song playback. Additional samples were also collected from these 4 MAPS stations. Two 3^rd^ rectrices were plucked from as many captured individuals as possible and individual body mass was recorded at time of capture. All feathers were stored in paper envelopes at room temperature. In the oil sands region, birds were captured and feathers were collected by KF, CG (banders-in-charge), and their banding staff. At Slave Lake, birds were captured and feather samples were collected by RK (bander-in-charge), LSLBO banding staff, and volunteers.

**Table 1:** Locations, habitat descriptions, mean *δ*^2^H_f_ (± SD), and number of feather samples (n) collected from 36 capture locations in northeastern Alberta, Canada.

Station	Lat	Long	Habitat (100 m radius)^a^	Habitat (Station)	Wetland area (m^2^)	*δ*^2^H_f_^b^ (± SD)	n
1	56.981	-111.619	Broadleaf	Exposed Land	13750	-136 (± 23)	14
2^c^	57.006	-111.608	Coniferous	Exposed Land	5000	-159	1
3^c^	57.022	-111.637	Mixedwood	Exposed Land	7500	-147 (± 17)	32
4	57.169	-111.536	Wetland	Wetland-Shrub	13125	-155 (± 3)	2
5	57.044	-111.538	Mixedwood	Exposed Land	2500	-147 (± 15)	4
6	57.247	-111.595	Mixedwood	Wetland-Shrub	0	-146	1
7	55.616	-111.041	Coniferous	Broadleaf	8125	-130 (± 27)	4
8	57.169	-111.038	Wetland	Water	21875	-150 (± 3)	4
9	57.080	-111.689	Wetland	Shrub-Tall	21875	-136 (±13)	10
10	57.248	-111.735	Mixedwood	Water	20625	-138 (±14)	14
11	57.240	-111.735	Mixedwood	Coniferous	15625	-144 (± 4)	5
12	56.201	-110.893	Mixedwood	Coniferous	13750	-123 (± 26)	4
13	56.997	-111.554	Mixedwood	Broadleaf	0	-136	1
14	57.382	-111.885	Broadleaf	Wetland-Shrub	8750	-138 (± 8)	5
15	57.393	-111.983	Mixedwood	Broadleaf	11250	-143 (± 4)	7
16	56.419	-111.375	Broadleaf	Wetland-Treed	5625	-149 (± 4)	2
17	56.697	-111.398	Broadleaf	Coniferous	9375	-140	1
18	57.301	-111.217	Mixedwood	Broadleaf	15000	-142 (± 11)	2
19	57.209	-111.692	Mixedwood	Broadleaf	23750	-145 (± 7)	4
20	55.536	-110.889	Broadleaf	Coniferous	10000	-136	1
21	57.313	-111.212	Coniferous	Broadleaf	10000	-144 (± 6)	4
22	57.181	-111.584	Broadleaf	Coniferous	625	-142	1
23	57.197	-111.046	Broadleaf	Wetland-Shrub	0	-137 (± 10)	6
24	56.916	-111.458	Broadleaf	Wetland-Shrub	11250	-150 (± 10)	12
25	56.924	-111.503	Coniferous	Broadleaf	1250	-149 (± 7)	5
26	57.155	-111.063	Broadleaf	Coniferous	0	-141 (± 4)	7
27^c^	57.040	-111.596	Broadleaf	Exposed Land	1250	-162 (± 10)	5
28	55.390	-110.744	Broadleaf	Coniferous	0	-138	1
29	55.571	-110.903	Broadleaf	Wetland-Treed	625	-124 (± 22)	2
30	56.190	-110.973	Broadleaf	Broadleaf	0	-141	1
31	57.198	-111.531	Broadleaf	Broadleaf	4375	-146 (± 3)	3
32	57.257	-111.041	Broadleaf	Coniferous	5000	-148 (± 7)	5
33^d^	55.429	-114.829	Coniferous	Wetland-Shrub	1250	-150 (± 9)	22

Habitat information (i.e. dominant land cover type at point location [station] and within 100 m radius and wet area within a 100 m radius) was extracted from the Earth Observation for the Sustainable Development of Forests EOSD [33].

^a^ Surrounding forest canopy dominated by: Broad Leaf = leafy deciduous vegetation, Coniferous = spruce, pine and needled vegetation, Mixed Wood = both leafy and coniferous vegetation, and Wetland = tall vegetation limited in peat or grassy water dominated terrain.

^b^ Mean *δ*^2^H_f_ values for all feather samples collected at a given station.

^c^ Banding station within a reclaimed site.

^d^ Banding stations and mist netting sites from LSLBO were considered a single banding station (central location is provided) given they were all within a 1 km radius.

Only feathers from males and females of at least two years old (i.e. after-second year individuals; ASY) were used for isotope analysis. These individuals were selected based on the higher site fidelity reported in adult songbirds compared to first-year breeders [31, 34]. Also, adult Neotropical migratory passerines generally undergo complete pre-basic molts, including the replacement of primaries and rectrices, at or near their breeding site [13]. Thus, for most species, our feather samples should have reflected the isotopic signature incorporated at our study site in the previous breeding season. Individuals from LSLBO were aged and sexed by the banders-in-charge, while those from the oil sands region were aged and sexed by the banders-in-charge and photos were reviewed by P. Pyle (Institute for Bird Populations; http://www.birdpop.org/). We further determined age of some *S*. *aurocapilla* by quantifying the wear pattern of the 3^rd^ rectrices [35]. Feather samples were required from a minimum of nine individuals for a species to be considered. For each location, we also identified the dominant land cover type at the point level and in a 100 m radius (exposed land, wetland-shrub, wetland-treed, shrub-tall, broadleaf, coniferous and water), and wet area (ha) within a 100 m radius using the Earth Observation for Sustainable Development of Forests (EOSD; [33]). For each species, we assigned dietary categories (i.e. insectivore vs. omnivore), forage substrate (i.e. upper canopy, lower canopy/shrub, and ground), and nest substrate (i.e. agriculture, bogs, tree / shrub swamp, coniferous, deciduous, early successional, marsh, mixedwood, and open stands) using the Avian Life History Information Database (hereafter “ALHD”; http://www.on.ec.gc.ca/wildlife/wildspace/project.cfm; Table 2).

**Table 2:** Number of feather samples (n) collected from 21 songbird species breeding in northern Alberta, Canada.

Scientific Name	Common Name (4-letter code)	N	Forage Substrate^a^	Diet^b^	Nesting Substrate
*Empidonax alnorum*	Alder Flycatcher (ALFL)	15	A	I	Treed/shrubby swamp
*Setophaga ruticilla*	American Redstart (AMRE)	14	LCS	I	Deciduous Woodland
*Cardellina canadensis*	Canada Warbler (CAWA)	14	LCS	I	Deciduous Woodland
*Spizella pallida*	Clay-coloured Sparrow (CCSP)	15	G	O	Coniferous Woodland
*Bombycilla cedrorum*	Cedar Waxwing (CEDW)	12	A	I	Open Woodland
*Spizella passerina*	Chipping Sparrow (CHSP)	15	G	O	Open Woodland
*Geothlypis trichas*	Common Yellowthroat (COYE)	9	LCS	I	Marsh
*Empidonax minimus*	Least Flycatcher (LEFL)	15	A	I	Deciduous Woodland
*Melospiza lincolnii*	Lincoln's Sparrow (LISP)	12	G	O	Bogs
*Setophaga magnolia*	Magnolia Warbler (MAWA)	11	LCS	I	Mixed Woodland
*Geothlypis philadelphia*	Mourning Warbler (MOWA)	13	G	I	Open Woodland
*Setophaga coronata*	Yellow-rumped Warbler (YRWA)	13	LCS	I	Coniferous Woodland
*Seiurus aurocapilla*	Ovenbird (OVEN)	11	G	I	Deciduous Woodland
*Vireo olivaceus*	Red-eyed Vireo (REVI)	15	UC	I	Deciduous Woodland
*Passerculus sandwichensis*	Savannah Sparrow (SAVS)	14	G	O	Agricultural
*Melospiza melodia*	Song Sparrow (SOSP)	12	LCS	O	Early Successional
*Catharus ustulatus*	Swainson's Thrush (SWTH)	14	G	O	Mixed Woodland
*Oreothlypis peregrina*	Tennessee Warbler (TEWA)	14	UC	I	Bogs
*Tachycineta bicolor*	Tree Swallow (TRES)	15	A	I	Treed/shrubby swamp
*Zonotrichia albicollis*	White-throated Sparrow (WTSP)	13	G	O	Early Successional
*Setophaga petechia*	Yellow Warbler (YEWA)	13	LCS	I	Early Successional

Life history summary was provided by Avian Life History Information Database (http://www.on.ec.gc.ca/wildlife/wildspace/project.cfm).

^a^ Description of Forage substrate: A = aerial, G = ground, LCS = lower canopy / shrub, UC = upper canopy.

^b^ Description of diet types: I = Insectivore, O = Omnivore.

Fieldwork was conducted in accordance with the Canadian Council for Animal Care and the permit for this study was approved by the University of Alberta Animal Care Committee (permit # AUP00000100). Federal and provincial bird banding and feather collection permits for this study were approved by the Canadian Wildlife Service and Alberta Environment and Sustainable Resource Development, respectively.

### Stable isotope analysis

Surface oils were removed from all feathers by using a 2:1 chloroform:methanol solution. The central vane of each feather was weighed (350 ± 20 μg) and samples were secured in silver capsules. Isotopic analysis was conducted at the Colorado Plateau Stable Isotope Laboratory at the Northern Arizona University for 2011 samples and the Stable Isotope Hydrology and Ecology Laboratory of Environment Canada for 2013 samples. Samples were exposed to high temperature (1350°C) flash pyrolysis and the separated H_2_ pulses were used to measure *δ*^2^H_f_ by continuous-flow isotope-ratio mass spectrometry (CF-IRMS). For both laboratories, we used the comparative equilibration approach with the same in-house keratin working standards (KHS [–54.1‰], SPK [–121.6‰], CBS [–197‰]) to account for exchangeable hydrogen in keratins where *δ*^2^H of nonexchangeable H was established [36]. Thus we are confident that isotope results are comparable within measurement error between the two laboratories (see [8]). Results were expressed for non-exchangeable H delta notation (*δ*^2^H_f_) in units of per mil (‰) and the analytical error, based on within-run replicates of keratin reference standards (n = 5 per run) was ±2‰. Results are reported relative to the Vienna Standard Mean Ocean Water—Standard Light Antarctic Precipitation (VSMOW-SLAP) scale.

### Statistical analyses

Linear mixed models were used to explore how *δ*^2^H_f_ values from individuals of the selected species captured in 2013 were influenced by species, individual, and land cover factors (fixed effects) and phylogenetic relationships across species (random effects). A Multivariate Normal distribution was specified for each model where fixed effects are part of the mean specification, while random effects are part of the covariance structure. The response variable (*y*_*ij*_) was *δ*^2^H_f_ for individual *i* (*i* = 1…*n*) of species *j* (*j* = 1…*s*). We examined 15 different fixed effects as possible explanatory factors for *δ*^2^H_f_ and compared them to a (0) null (intercept only) model. The fixed effects included species (1—species, 2—forage substrate [ALHD], 3—diet [ALHD], and 4—nest substrate [ALHD]), banding station (5—land cover type at banding station [EOSD], 6—dominant land cover type [EOSD], 7—wetland area in 100 m radius of each banding station [EOSD], and 8—latitude), individual (9—linear and 10—quadratic individual mass, and 11—sex), and interactive effects (12—forage substrate × diet, 13—forage substrate × land cover, 14—nest substrate × diet, and 15—forage substrate × diet × nest substrate; Table 3). We used the Akaike Information Criterion corrected for small sample sizes (AICc; [37]) to select the most parsimonious model describing variation in *δ*^2^H_f_ among feather samples.

**Table 3:** Candidate models considered in this study.

Model	Fixed Effects^a^	Set-Hom	Set-Het	Scale
0	Null Model	σ^2^, λ	σ^2^_j,_ λ	-
1	Species	σ^2^, λ	σ^2^_j,_ λ	Species
2	ForSub	σ^2^, λ	σ^2^_j,_ λ	Species
3	Diet	σ^2^, λ	σ^2^_j,_ λ	Species
4	NestSub	σ^2^, λ	σ^2^_j,_ λ	Species
5	LandStation	σ^2^, λ	σ^2^_j,_ λ	Station
6	Land100m	σ^2^, λ	σ^2^_j,_ λ	Station
7	WetArea	σ^2^, λ	σ^2^_j,_ λ	Station
8	Latitude	σ^2^, λ	σ^2^_j,_ λ	Station
9	IndMass	σ^2^, λ	σ^2^_j,_ λ	Individual
10	IndMass^2^	σ^2^, λ	σ^2^_j,_ λ	Individual
11	Sex	σ^2^, λ	σ^2^_j,_ λ	Individual
12	ForSub × Diet	σ^2^, λ	σ^2^_j,_ λ	Species
13	ForSub × Habitat	σ^2^, λ	σ^2^_j,_ λ	Species
14	NestSub × Diet	σ^2^, λ	σ^2^_j,_ λ	Species
15	ForSub × Diet × NestSub	σ^2^, λ	σ^2^_j,_ λ	Species

For each model subset, the variance was estimated as homoscedastic, where *δ*^2^H_f_ variances are constant across all species (Set-Hom; σ^2^) or heteroscedastic, where *δ*^2^H_f_ variances are flexible across all species (Set-Het; σ^2^_j_). All candidate models estimated strength of the phylogenetic correlation (λ [38]). Each model also described processes acting at different levels (i.e. Individual, Species or Station).

^a^ Description of variables: ForSub = ground vs non-ground foragers, Diet = insectivore vs omnivore, NestSub = typical nesting habitat, LandStation, Land100m and WetArea = land cover at station, dominant land cover within 100 m radius, and wetland are within 100 m according to EOSD [33], respectively, IndMass and IndMass^2^ = linear and quadratic individual mass at time of capture, respectively.

Residual variance (σ^2^) was included as a random effect in our 15 models and we divided each model into two subsets where variance in *δ*^2^H_f_ among species was estimated as homoscedastic (variation among species was assumed equal [σ^2^]; Hom1—Hom15) and heteroskedastic (the assumption of variance equality among species was relaxed [σ^2^_j_]; Het1 -Het15). To examine the phylogenetic correlation in *δ*^2^H_f_, we estimated a multiplier of the off-diagonal elements in the Multivariate Normal covariance matrix defined as λ [38, 39]. A value of λ = 0 indicated evolution of traits independent of phylogeny, while value of λ = 1 indicated traits evolving according to Brownian motion. Values <1 indicated that phylogeny effect was weaker than expected under the Brownian model. The covariance between two observations (*y*_*ij*_, *y*_*ik*_) and corresponding Mutivariate Normal means (*μ*_*ij*_, *μ*_*ik*_) was defined as cov(*y*_*ij*_, *y*_*ij*_) = *σ*_*j*_ *σ*_*k*_ *t*_*jk*_ *λ*, where *t*_*jk*_ is proportional to the shared evolutionary path length between species *j* and *k*.

Shared evolutionary path lengths were estimated using 5000 pseudo-posterior trees based on genetic data [40]. A phylogenetic correlation matrix [41] was constructed for each of the 5000 trees. Elements of the correlation matrix were defined as lengths of branches shared between species based on mean node heights [42, 43]. The different number of observations per species meant we could not apply existing phylogenetic mixed model software which uses only 1 observation per species. Therefore, we implemented a maximum likelihood estimating procedure for Multivariate Normal mixed models using Markov-chain Monte Carlo methods and a data cloning algorithm [44] using the ‘dclone’ R package [45] and JAGS [46].

A two-way ANOVA was used to test for a Year (2011 and 2013) × Species (Ovenbird, Swainson's Thrush, American Redstart and Yellow-rumped Warbler) interaction effect on *δ*^2^H_f_. All analyses were conducted using R version 3.1.0 [47]. Results are presented as mean *δ*^2^H_f_ ± standard deviation (SD) unless specified otherwise. We also extracted δ^2^H_p_ values for our study area from the isoscape provided by Bowen et al. [48] (see also IsoMap, isomap.org). Values were expressed as the annual mean growing season (i.e. months where mean monthly temp > 0°C) and used to estimate δ^2^H_f_ for omnivores and insectivores following Hobson et al. [17].

## Results

**Fig 1:**
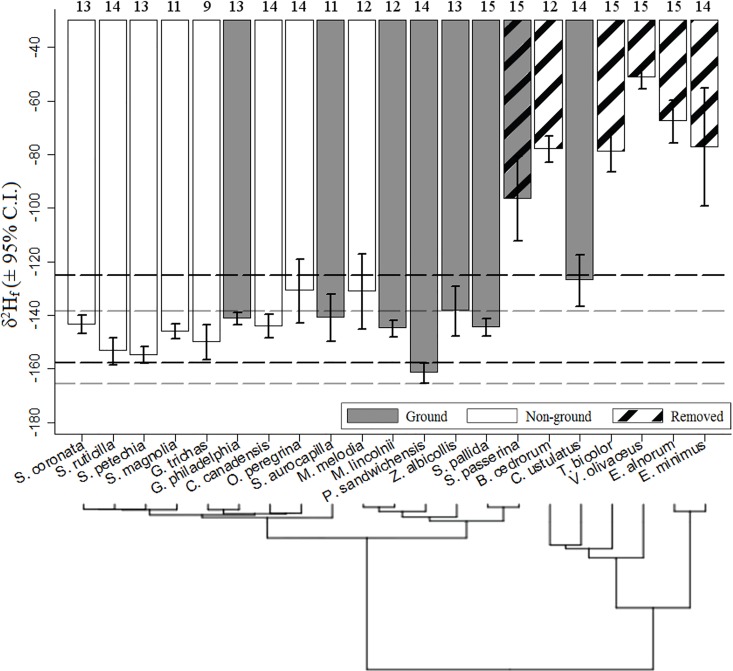
Mean *δ*^2^H_f_ (± 95% confidence intervals) for 278 ASY individuals of 21 songbird species breeding in northern Alberta, Canada, in 2013. The dashed black and grey horizontal lines indicate the standard deviation of the residuals from Hobson et al. [17]'s *δ*^2^H_f_ transfer functions for ground (Ground) and non-ground foraging (Non-ground) songbirds, respectively. The phylogenetic tree was derived from 5000 pseudo-posterior trees; shorter branch lengths occur between species that are more closely related with each other. Also indicated are the sample size for each species and the six species removed from further analyses because they were believed to have molted their tail feathers on the wintering grounds or during migration.

**Fig 2:**
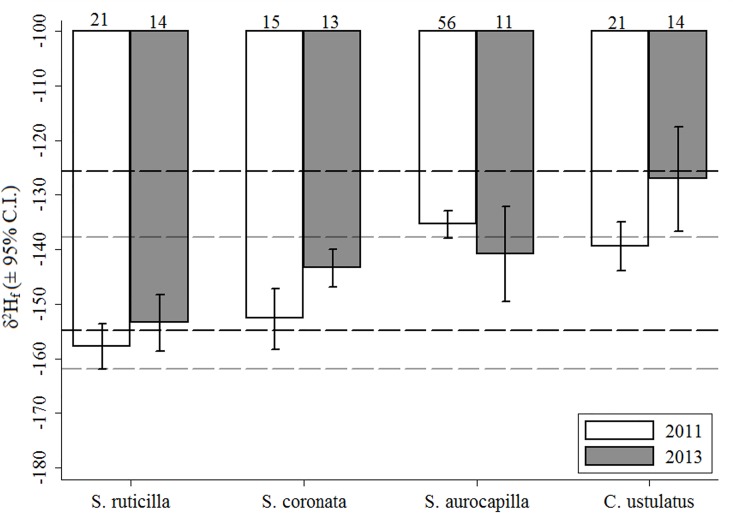
Mean *δ*^2^H_f_ (± 95% confidence intervals) for 4 songbird species breeding in northern Alberta, Canada, in 2011 and 2013. The black and grey horizontal lines indicate standard deviation of the residuals from Hobson et al. [17]’s *δ*^2^H_f_ transfer functions for ground (Ground) and non-ground foraging (Non-ground) songbirds, respectively. Sample size is indicated above each bar.

We analyzed *δ*^2^H_f_ from 278 ASY individuals of 21 songbird species captured in 2013 (Table 2). Mean values for most species (15 species) ranged from -127‰ (Swainson's Thrush) to -162‰ (Savannah Sparrow), while six species (Chipping Sparrow, Tree Swallow, Red-eyed Vireo, Least Flycatcher, Cedar Waxwing, Alder Flycatcher) had higher mean *δ*^2^H_f_ (-97‰ to -51‰; Fig 1). Combined with large variation around mean values for these six species, these results suggested that rectrices from these individuals were grown on the wintering ground or during migration. These results, at the exception of those for Cedar Waxwing, are consistent with previous accounts [13, 49] and these six species were removed from further analyses. The remaining samples included 192 ASYs (43 females and 149 males) from 15 species. We also report *δ*^2^H_f_ for 113 ASY of the 4 species captured in 2011 (Fig 2).

Standard deviations were smallest for Mourning Warbler (± 4‰) and largest for Song Sparrow (± 22‰; Fig 1). The 95% confidence intervals for species-specific mean *δ*^2^H_f_ overlapped with the predicted range of *δ*^2^H_f_ values from the transfer functions of Hobson et al. [16] for ground and non-ground foragers in our study area (Fig 1). Mean values of ground foragers and non-ground foragers (irrespective of species) were -143 ± 14‰ and -144 ± 15‰, respectively, both 95% confidence intervals overlap with the respective *δ*^2^H_f_ estimates from Hobson et al. [17] for our study area. Males and females had similar mean and standard deviation in *δ*^2^H_f_ (-143 ± 14‰ and -146 ± 11‰, respectively).

**Fig 3:**
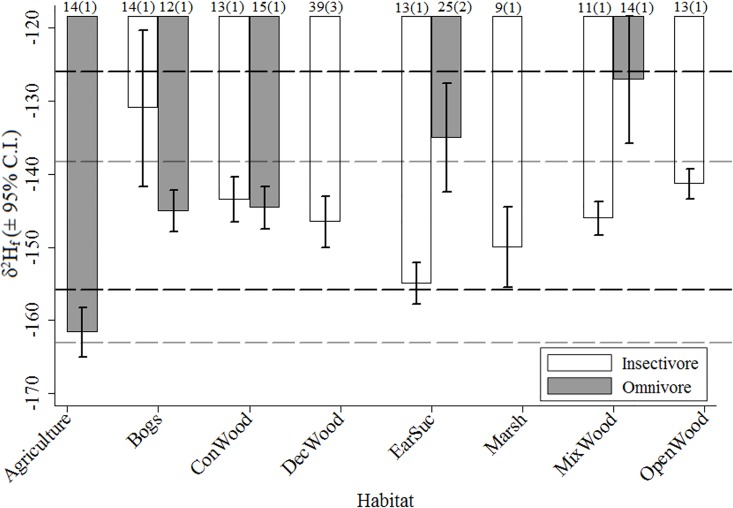
Mean *δ*^2^H_f_ (± 95% confidence intervals) for 15 songbird species grouped by diet and habitat association. Individuals were captured in 2013 and assumed to have molted their feathers prior to fall migration the previous year. Means values are reported by diet and habitat association according to the Avian Life History Information Database (http://www.on.ec.gc.ca/wildlife/wildspace/project.cfm). The black and grey horizontal lines indicate standard deviation of the residuals from Hobson et al. [17]’s *δ*^2^H_f_ transfer functions for ground (Ground) and non-ground foraging (Non-ground) songbirds, respectively. Also indicated is the number of samples for each category and species in brackets.

Models incorporating a species-specific variance parameter (heteroscedasticity) performed better than the homoscedastic model subsets (S1 and S2 Tables). Our top model, included species as predictor (Het1; wAICc = 0.74). The second-best ranked model included a nest substrate × diet interaction (Het14; ΔAICc = 3.02; wAICc = 0.16). The next two models including nest substrate x diet x forage substrate and nest substrate as a single effect received little support (Het15 and Het4; ΔAICc > 5), but were the only other models with a wAICc > 0.001. The four top-ranked models included only species-level predictors and provided little evidence for an important effect of phylogeny (λ ≤ 0.29), land cover (wAICc < 0.001), or individual (wAICc < 0.001) in explaining variation in *δ*^2^H_f_ (S1 and S2 Tables). Mean *δ*^2^H_f_ ranged from -135 ± 16‰ (mixedwood) to -162 ± 6‰; (agriculture) across nest substrate (Fig 3) and was -142 ± 17‰ and -145 ± 12‰ between omnivores and insectivores, respectively (Fig 3). Lastly, there was a significant species × year interaction effect (F_7, 157_ = 19.9, *p* < 0.001). Three of the four species had higher *δ*^2^H_f_ values in 2013 compared to 2011 (4‰, 9‰, and 12‰, respectively), while the Ovenbird had lower *δ*^2^H_f_ values in 2013 compared to 2011 (-6‰; Fig 2).

## Discussion

This study demonstrates important interspecific differences in *δ*^2^H_f_ among songbird species breeding in the same region. Species was the strongest predictor of the variation in *δ*^2^H_f_ among individuals followed by a nesting substrate × diet composition interaction effect. Limited support was found for single effect models that included life history traits, individual (sex and body mass), or land cover predictors. Some studies have found support for these predictors [17, 23, 50], but they did not compare the relative importance of many predictors for different levels of organization. We also showed that differences in *δ*^2^H_f_ between years are not always consistent among species. Our results provide important insights regarding different sources of error being propagated in Bayesian assignment tests and suggest that the use of species- and year-specific *δ*^2^H_f_ isoscapes could improve the accuracy of assigned geographic origin of birds (see also [51], but see [52]).

Based on results from Hobson et al. [17], we predicted that ground foraging songbirds would have higher *δ*^2^H_f_ compared to those foraging in the upper- or lower-canopy. Mean *δ*^2^H_f_ values or corresponding 95% confidence intervals for the 15 focal species did indeed overlap with predicted values from the transfer functions of Hobson et al. [17] applied to our study area. However, models with foraging substrate as a single predictor received very little support (ΔAICc > 26.6). There was also no evidence that songbirds using open habitat or forest canopy during the nesting period had higher *δ*^2^H_f_ compared to those using forest understories. In temperate or boreal ecosystems, differences in ground temperature between closed and open habitats may not influence evapotranspiration rates and, ultimately, *δ*^2^H_f_. For example, Hache et al. [29] found no difference in *δ*^2^H_f_ between nestlings from recent selection harvesting (open) vs unharvested stands (closed). However, most adult songbirds start molting during the post-fledgling period and some mature forest specialists have been shown to aggregate in open-canopy clearcuts for concealment from predators during this period [30, 53]. Thus, many focal species might have used similar microhabitat and experienced similar temperatures and sun exposure during the molting period irrespective of their nesting substrate. Species-specific variation in timing and location of molt (e.g. [54]) could also result in important spatio-temporal variation in *δ*^2^H_f_ of ASY songbirds from a given region, but it is unclear to what extent resources consumed during the nesting period can be integrated in tissues that have grown during the post-fledging period (e.g. [55, 56]).

Birds with larger body sizes could have lower *δ*^2^H_f_ than smaller birds because larger individuals or species lose proportionately less of their total body water to evaporative processes [21, 22]. However, there was no evidence in our study to suggest body size was important for *δ*^2^H_f_ in adult songbirds, but differences in body mass among individuals at the time of capture might not reflect differences during the molting period. Females also did not have different *δ*^2^H_f_ values than males [57] and variation around mean *δ*^2^H_f_ was only slightly lower for males than females. These results indicate that population level assignments can be generated irrespective of body size and sex of individuals.

Breeding dispersal distances appear to be species-specific [57, 58] and would often occur over relatively short distances. Variation in mean *δ*^2^H_f_ can be used as a proxy for breeding dispersal rate and distance, i.e. species with larger variation might have a higher proportion of individuals that experienced breeding dispersal movements and/or dispersed over larger spatial extents. The relatively small variation around mean *δ*^2^H_f_ reported for all focal species in this study suggests that most dispersal events would have occurred within the isocline corresponding to predicted *δ*^2^H_f_ for our study area. However, differences in mean *δ*^2^H_f_ among species could also reflect population-level post-breeding movements (i.e. within year dispersal movements prior to fall migration) and molt occurring elsewhere in the species breeding range (e.g. [59]).

The better performance of heteroscedastic compared to homoscedastic variance models suggests an important species-specific component for future songbird assignment of geographic origins using *δ*^2^H_f_. Differences in variation around species-specific mean *δ*^2^H_f_ in a region might reflect interspecific differences in breeding dispersal rates, but also differences in niche breath. Habitat generalists use a broader range of land cover types which could result in greater variation in *δ*^2^H_f_ than habitat specialists. However, species-specific tolerance (i.e. the range of environmental conditions over which a species occurs; [60]) was not a good predictor of variation in *δ*^2^H_f_ for 13 of our focal species (R^2^ = 0.05; *p* = 0.46; S1 File). However, the interspecific variation in the range of environmental conditions used by forest songbirds might not be correlated with the range of *δ*^2^H_p_ values encountered by these species.

Studies have shown morphological traits to be more similar in closely related passerine species while for other traits, such as behavior and habitat associations, this might not be the case [24]. In this study, closely related species did not have more similar *δ*^2^H_f_ values than less related species. Our 15 focal species represented only 3 families (i.e. *Passeridae*, *Parulidae*, and *Turdidae*) and may have been too closely related to detect phylogenetic effects. We also expected a consistent difference in *δ*^2^H_f_ between years (2011 vs 2013) for the 4 focal species, but this effect was not observed. If year effects on *δ*^2^H_f_ were exclusively driven by differences in *δ*^2^H_p_ across years, we would expect a proportionate year effect across species breeding in the same region. Future studies should aim to identify the underlying mechanisms explaining this interaction effect. This could potentially be achieved by quantifying differences in *δ*^2^H_f_ for a broader range of species and years. Nonetheless, along with species-specific variation in *δ*^2^H_f_, this species × year interaction effect was another important factor and likely corresponds to a large proportion of the variance being propagated in Bayesian assignment tests [52].

We provided evidence suggesting that the most important source of error being propagated in assignment tests of geographic origin of birds is likely interspecific variation in *δ*^2^H_f._ However, we had limited success identifying the underlying mechanisms. Future studies should determine whether missing variables or interaction terms could explain interspecific variation in *δ*^2^H_f_ or more precisely determine where birds undergo the prebasic molt relative to breeding territories. A better understanding of the variation in *δ*^2^H_f_ within and across years (i.e. across a larger number of species and years) should provide important information about sources of error being propagated in assignments of geographic origin. Transfer functions to explicitly account for these sources of error would likely help generate more precise and accurate assignments and provide better information on migratory bird movements to inform full-cycle conservation strategies. However, our ability to integrate these sources of variance in assignment tests will depend on the objectives. For example, it might be unrealistic to expect that species- and year-specific *δ*^2^H_f_ isoscapes would be available over large spatial extent. Alternatively, it would be possible to document regional dispersal movements [3, 4]. Results from Vander Zanden et al. [52] also suggest that the need to use year-specific *δ*^2^H_f_ isoscapes would depend on the study region because the degree of inter-annual variation in *δ*^2^H_p_ differs among regions. It is also likely that we are close to identifying the limit of accuracy of this single isotope approach, highlighting the importance of combining multiple markers in multivariate assignment tests [59, 61–63].

Finally, there are a number of caveats that need to be considered in our study and future research is encouraged to investigate further potential mechanisms contributing to within-site variance in *δ*^2^H_f_ values. For example, it is still unclear how local hydrology interacts with biology by influencing relationships between *δ*^2^H values of foodwebs used by birds during the molt period and their subsequent *δ*^2^H_f_ values. At interior continental sites, there are extreme seasonal effects in precipitation *δ*^2^H values [64] and northern sites will also have potential contributions from snowmelt as well as growing-season precipitation. These factors can strongly influence resulting assumed environmental *δ*^2^H values experienced by molting birds. In northern regions where H from snowmelt contributes to the overall foodweb leading to bird feathers, the use of an amount-weighted mean annual precipitation *δ*^2^H value or an average corresponding to those months where *δ*^2^H from precipitation has the strongest influence on the foodweb may be more appropriate. Nonetheless, our study provides encouraging evidence that once isotopic variance is understood and accounted for, current assignment isotopic models provide a valuable means of propagating such variance into the most parsimonious depiction of origins for North American passerines.

## Supporting Information

S1 File Relationship between niche breadth and δ^2^H_f_ variation. (DOCX)

S1 Table Candidate models ranked according to AICc weights (wAICc). (DOCX)

S2 Table Parameter estimates from the top-ranked model used to explain variation in δ^2^H_f_ among 192 adult migratory songbirds (Het1). (DOCX)

## References

[1] Blancher P. Importance of Canada's Boreal Forest to Landbirds. Bird Studies Canada, 2003.

[2] Faaborg J, Holmes RT, Anders AD, Bildstein KL, Dugger KM, Gauthreaux SAJ, et al Recent advances in understanding migration systems of New World land birds. Ecological Monographs. 2010; 80:3–48.

[3] Studds CE, McFarland KP, Aubry Y, Rimmer CC, Hobson KA, Marra PP, et al Stable-hydrogen isotope measures of natal dispersal reflect observed population declines in a threatened migratory songbird. Diversity and Distributions. 2012; 18:919–930.

[4] Haché S, Hobson KA, Bayne EM, Van Wilgenburg SL, Villard M-A. Tracking natal dispersal in a coastal population of a migratory songbird using feather stable isotope (δ2H, δ34S) tracers. PloS ONE. 2014; 9:e94437 10.1371/journal.pone.0094437 24740314 PMC3989223

[5] Chabot AA, Hobson KA, Van Wilgenburg SL, McQuat GJ, Lougheed SC. Advances in linking wintering migrant birds to their breeding-ground origins using combined analyses of genetic and stable isotope markers. PloS ONE. 2012; 7:e43627 10.1371/journal.pone.0043627 22916285 PMC3423384

[6] Stutchbury BJM, Tarof SA, Done T, Gow E, Kramer PM, Tautin J, et al Tracking long-distance songbird migration by using geolocators. Science. 2009; 323:896 10.1126/science.1166664 19213909

[7] Webster MS, Marra PP, Haig SM, Bensch S, Holmes RT. Links between worlds: unraveling migratory connectivity. Trends in Ecology and Evolution. 2002; 17:76–83.

[8] Wassenaar LI. An Introduction to light stable isotopes for use in terrestrial animal migration Studies In: Hobson KA, Wassenaar LI, editors. Tracking Animal Migration with Stable Isotopes: Academic Press; 2008 pp. 21–44.

[9] Hobson KA. Applying isotopic methods to tracking animal movements In: Hobson KA, Wassenaar LI, editors. Tracking Animal Migration with Stable Isotopes: Academic Press; 2008 pp. 45–78.

[10] Bowen GJ. Isoscapes: spatial pattern in isotopic biogeochemistry. Annual Review of Earth and Planetary Sciences. 2010; 38:161–187.

[11] Clark RG, Hobson KA, Wassenaar LI. Geographic variation in the isotopic (δ D, δ13C, δ15N, δ34S) composition of feathers and claws from lesser scaup and northern pintail: implications for studies of migratory connectivity. Canadian Journal of Zoology. 2006; 84:1395–1401.

[12] Chamberlain CP, Blum JD, Holmes RT, Feng X, Sherry TW, Graves GR. The use of isotope tracers for identifying populations of migratory birds. Oecologia. 1997; 109:132–141. 10.1007/s004420050067 28307603

[13] Pyle P. Identification guide to North American birds, part 1 Colinas, CA: Slate Creek Press; 1997.

[14] Quinlan SP, Green DJ. Variation in deuterium (δD) signatures of Yellow Warbler *Dendroica petechia* feathers grown on breeding and wintering grounds. Journal of Ornithology. 2011; 152:93–101.

[15] Moran J, Wassenaar L, Finlay J, Hutcheson C, Isaac L, Wethington S. An exploration of migratory connectivity of the Rufous Hummingbird (*Selasphorus rufus*), using feather deuterium. Journal of Ornithology. 2013; 154:423–430.

[16] Pyle P. Age determination and molt strategies in North American alcids. Marine Ornithology. 2009; 37:219–226.

[17] Hobson KA, Van Wilgenburg SL, Wassenaar LI, Larson K. Linking hydrogen (δ2H) isotopes in feathers and precipitation: sources of variance and consequences for assignment to isoscapes. PloS ONE. 2012; 7:e35137 10.1371/journal.pone.0035137 22509393 PMC3324428

[18] Birchall J, O'Connell TC, Heaton THE, Hedges REM. Hydrogen isotope ratios in animal body protein reflect trophic level. Journal of Animal Ecology. 2005; 74:877–881.

[19] Vander Zanden HB, Wunder MB, Hobson KA, Wilgenburg SL, Wassenaar LI, Welker JM, et al Space‐time tradeoffs in the development of precipitation‐based isoscape models for determining migratory origin. Journal of Avian Biology. 2015; 46:658–667.

[20] McKechnie AE, Wolf BO, Martinez del Rio C. Deuterium stable isotope ratios as tracers of water resource use: an experimental test with rock doves. Oecologia. 2004; 140:191–200. 10.1007/s00442-004-1564-9 15185137

[21] Dawson WR. Evaporative losses of water by birds. Comparative Biochemistry and Physiology Part A: Physiology. 1982; 71:495–509. 10.1016/0300-9629(82)90198-0 6124338

[22] Rezende EL, López-Calleja MV, Bozinovic F. Standard and comparative energetics of a small avian herbivore (*Phytotoma rara*). The Auk. 2001; 118:781–785.

[23] Betini GS, Hobson KA, Wassenaar LI, Norris DR. Stable hydrogen isotope (δD) values in songbird nestlings: effects of diet, temperature, and body size. Canadian Journal of Zoology. 2009; 87:767–772.

[24] Böhning-Gaese K, Oberrath R. Phylogenetic effects on morphological, life-history, behavioural and ecological traits of birds. Evolutionary Ecology Research. 1999; 1:347–364.

[25] Pienaar J, Ilany A, Geffen E, Yom‐Tov Y. Macroevolution of life‐history traits in passerine birds: adaptation and phylogenetic inertia. Ecology Letters. 2013; 16:571–576. 10.1111/ele.12077 23489254

[26] Sessions AL, Burgoyne TW, Schimmelmann A, Hayes JM. Fractionation of hydrogen isotopes in lipid biosynthesis. Organic Geochemistry. 1999; 30:1193–1200.

[27] Sessions AL, Hayes JM. Calculation of hydrogen isotopic fractionations in biogeochemical systems. Geochimica et Cosmochimica Acta. 2005; 69:593–597.

[28] Langin KM, Reudink MW, Marra PP, Norris DR, Kyser TK, Ratcliffe LM. Hydrogen isotopic variation in migratory bird tissues of known origin: implications for geographic assignment. Oecologia. 2007; 152:449–457. 10.1007/s00442-007-0669-3 17370093

[29] Hache S, Hobson KA, Villard MA, Bayne EM. Assigning birds to geographic origin using feather hydrogen isotope ratios (δ2H): importance of year, age, and habitat. Canadian Journal of Zoology. 2012; 90:722–728.

[30] Vitz AC, Rodewald AD. Can regenerating clearcuts benefit mature-forest songbirds? An examination of post-breeding ecology. Biological Conservation. 2006; 127:477–486.

[31] Greenwood PJ, Harvey PH. The natal and breeding dispersal of birds. Annual Review of Ecology and Systematics. 1982; 13:1–21.

[32] DeSante DF, Burton KM, Velez P, Froehlich D, Kaschube D. Maps Manual 2011 Protocol: instruction for the establishment and operation of constant-effort bird-banding stations as part of the monitoring avian productivity and survivorship (MAPS) program Point Reyes Station, CA: 2011.

[33] Wunder MB, Ryan Norris D. Analysis and Design for Isotope-Based Studies of Migratory Animals. Tracking animal migration with stable isotopes. 22008. pp. 107–128.

[34] Cooper CB, Daniels SJ, Walters JR. Can we improve estimates of juvenile dispersal distance and survival? Ecology. 2008; 89:3349–3361. 19137942 10.1890/08-0315.1

[35] Donovan TM, Stanley CM. A new method of determining ovenbird age on the basis of rectrix shape Journal of Field Ornithology. 1995; 66:247–252.

[36] Wassenaar L, Hobson K. Comparative equilibration and online technique for determination of non-exchangeable hydrogen of keratins for use in animal migration studies. Isotopes in Environmental and Health Studies. 2003; 39:211–217. 10.1080/1025601031000096781 14521282

[37] Burnham KP, Anderson DR. Model selection and multimodel inference: a practical information-theoretic approach: Springer Science & Business Media; 2002.

[38] Freckleton RP, Harvey PH, Pagel M. Phylogenetic analysis and comparative data: A test and review of evidence. The American Naturalist. 2002; 160:712–726. 10.1086/343873 18707460

[39] Pagel M. Inferring the historical patterns of biological evolution. Nature. 1999; 401:877–884. 10.1038/44766 10553904

[40] Jetz W, Thomas G, Joy J, Hartmann K, Mooers A. The global diversity of birds in space and time. Nature. 2012; 491:444–448. 10.1038/nature11631 23123857

[41] Martins EP, Hansen TF. Phylogenies and the comparative method: A general approach to incorporating phylogenetic information into the analysis of interspecific data. The American Naturalist. 1997; 149:646–667.

[42] Garland T Jr., Ives AR. Using the past to predict the present: confidence intervals for regression equations in phylogenetic comparative methods. The American Naturalist. 2000; 155:346–364. 10.1086/303327 10718731

[43] Rohlf FJ. Comparative methods for the analysis of continuous variables: geometric interpretations. Evolution. 2001; 55:2143–2160. 11794776 10.1111/j.0014-3820.2001.tb00731.x

[44] Lele SR, Nadeem K, Schmuland B. Estimability and likelihood inference for generalized linear mixed models using data cloning. Journal of the American Statistical Association. 2010; 105:1617–1625.

[45] Sólymos P. dclone: Data Cloning in R. The R Journal. 2010; 2:29–37.

[46] Plummer M. JAGS Version 3.4.0 manual, 2014. Available: http://mcmc-jags sourceforgenet. 2014.

[47] R Core Team. R: A language and environment for statistical computing. R Foundation for Statistical Computing, Vienna Austria. 2014; Available: http://www.R-project.org/.

[48] Bowen GJ, Wassenaar LI, Hobson KA. Global application of stable hydrogen and oxygen isotopes to wildlife forensics. Oecologia. 2005; 143:337–348. 10.1007/s00442-004-1813-y 15726429

[49] Pyle P, Leitner WA, Lozano-Angulo L, Avilez-Teran F, Swanson H, Limón EG, et al Temporal, Spatial, and annual variation in the occurrence of molt-migrant passerines in the mexican monsoon region. The Condor. 2009; 111:583–590.

[50] Fraser KC, McKinnon EA, Diamond AW, Chavarría L. The influence of microhabitat, moisture and diet on stable-hydrogen isotope variation in a Neotropical avian food web. Journal of Tropical Ecology. 2011; 27:563–572.

[51] van Dijk JG, Meissner W, Klaassen M. Improving provenance studies in migratory birds when using feather hydrogen stable isotopes. Journal of Avian Biology. 2014; 45:103–108.

[52] Vander Zanden HB, Wunder MB, Hobson KA, Van Wilgenburg SL, Wassenaar LI, Welker JM, et al Contrasting assignment of migratory organisms to geographic origins using long‐term versus year‐specific precipitation isotope maps. Methods in Ecology and Evolution. 2014; 5:891–900.

[53] Streby HM, Peterson SM, McAllister TL, Andersen DE. Use of early-successional managed northern forest by mature-forest species during the post-fledging period. The Condor. 2011; 113:817–824.

[54] Flockhart DTT. Timing of events on the breeding grounds for five species of sympatric warblers. Journal of Field Ornithology. 2010; 81:373–382.

[55] Legagneux P, Fast PLF, Gauthier G, Bêty J. Manipulating individual state during migration provides evidence for carry-over effects modulated by environmental conditions. Proceedings of the Royal Society of London B: Biological Sciences. 2012; 279:876–883. 10.1098/rspb.2011.1351 PMC3259927 21865256

[56] McKinnon EA, Fraser KC, Diamond AW, Rimmer CC, Townsend JM. Stable-hydrogen isotope turnover in red blood cells of two migratory thrushes: application to studies of connectivity and carry-over effects. Journal of Field Ornithology. 2012; 83:306–314.

[57] Clarke AL, Sæther B-E, Røskaft E. Sex biases in avian dispersal: A reappraisal. Oikos. 1997; 79:429–438.

[58] Paradis E, Baillie SR, Sutherland WJ, Gregory RD. Patterns of natal and breeding dispersal in birds. Journal of Animal Ecology. 1998; 67:518–536.

[59] Hobson KA, Kardynal KJ. Western Veeries use an eastern shortest-distance pathway: New insights to migration routes and phenology using light-level geolocators. The Auk. 2015; 132:540–550.

[60] Mahon CL, Holloway G, Sólymos P, Cumming SG, Bayne EM, Schmiegelow FKA, et al Community structure and niche characteristics of upland and lowland western boreal birds at multiple spatial scales. Forest Ecology and Management. 2016; 361:99–116.

[61] Rushing CS, Ryder TB, Saracco JF, Marra PP. Assessing migratory connectivity for a long-distance migratory bird using multiple intrinsic markers. Ecological Applications. 2014; 24:445–456. 24834732 10.1890/13-1091.1

[62] Crysler ZJ, Ronconi RA, Taylor PD. Differential fall migratory routes of adult and juvenile Ipswich Sparrows (*Passerculus sandwichensis princeps*). Movement Ecology. 2016; 4:1–8. 26819707 10.1186/s40462-016-0067-8 PMC4729120

[63] Rundel CW, Wunder MB, Alvarado AH, Ruegg KC, Harrigan R, Schuh A, et al Novel statistical methods for integrating genetic and stable isotope data to infer individual-level migratory connectivity. Molecular Ecology. 2013; 22:4163–4176. 10.1111/mec.12393 23906339

[64] Clark ID, Fritz P. Environmental isotopes in hydrogeology: CRC press, London.; 1997.

