# Correction: Within-Site Variation in Feather Stable Hydrogen Isotope (δ^2^H_f_) Values of Boreal Songbirds: Implications for Assignment to Molt Origin

**DOI:** 10.1371/journal.pone.0172619

**Published:** 2017-02-15

**Authors:** Cameron J. Nordell, Samuel Haché, Erin M. Bayne, Péter Sólymos, Kenneth R. Foster, Christine M. Godwin, Richard Krikun, Peter Pyle, Keith A. Hobson

The following information is missing from the Funding section: This study was supported by research grants from the Natural Sciences and Engineering Research Council of Canada (NSERC) to E.M.B. and K.A.H., Conservation Funds from the Alberta Conservation Association to E.M.B., an operating grant from Environment Canada to K.A.H., a NSERC Postgraduate Scholarship, Queen Elizabeth II Graduate Scholarship (University of Alberta), grants from the Canadian Circumpolar Institute and the Northern Scientific Program (University of Alberta), and a Dissertation Fellowship (University of Alberta) to S.H. Funding in support of the Boreal MAPS in the Oil Sands Program was provided by Syncrude Canada Ltd., Hammerstone Corporation, Canadian Natural Resources Limited., Cenovus Energy, ConocoPhillips Canada, Devon Energy, Husky Oil Operations Ltd., Imperial Oil Ltd., Suncor Energy, and TOTAL E&P Canada.

The following information is missing from the Competing Interests section: The authors received funding in support of the Boreal MAPS in the Oil Sands Program from Syncrude Canada Ltd., Hammerstone Corporation, Canadian Natural Resources Limited, Cenovus Energy, ConocoPhillips Canada, Devon Energy, Husky Oil Operations Ltd., Imperial Oil Ltd., Suncor Energy, and TOTAL E&P Canada, all commercial companies, for this study.
