# Supplementary material for: Within-Site Variation in Feather Stable Hydrogen Isotope (δ2Hf) Values of Boreal Songbirds: Implications for Assignment to Molt Origin
Source: PLoS One. 2016 Nov 2;11(11):e0163957. doi: 10.1371/journal.pone.0163957 (PMC5091831; doi:10.1371/journal.pone.0163957)

**S1 File. Results from a linear regression model testing for effects of niche breadth (Tolerance) on δ^2^H_f_.** We tested for an effect of tolerance on species-specific variation in δ^2^H_f_ using a linear regression model. Tolerance estimates for each species was provided by Mahon et al. [57]. The authors define tolerance (or niche breath) as the range of environment conditions or the length of the environmental gradient where a species occurs. Low tolerance means that a species occurs across a limited range of environmental conditions (specialist species) and high tolerance means that a species occurs across a wide range of environmental conditions (generalist species). Tolerance values were estimated for 13 of our focal species based on avian point count data and land cover information collected in the Joint Oil Sands Monitoring area (Alberta, Canada) in 2012 and 2013. Values were not available for Song Sparrow and Savannah Sparrow. Species-specific variance estimates were estimated from our top ranked model (Het1).


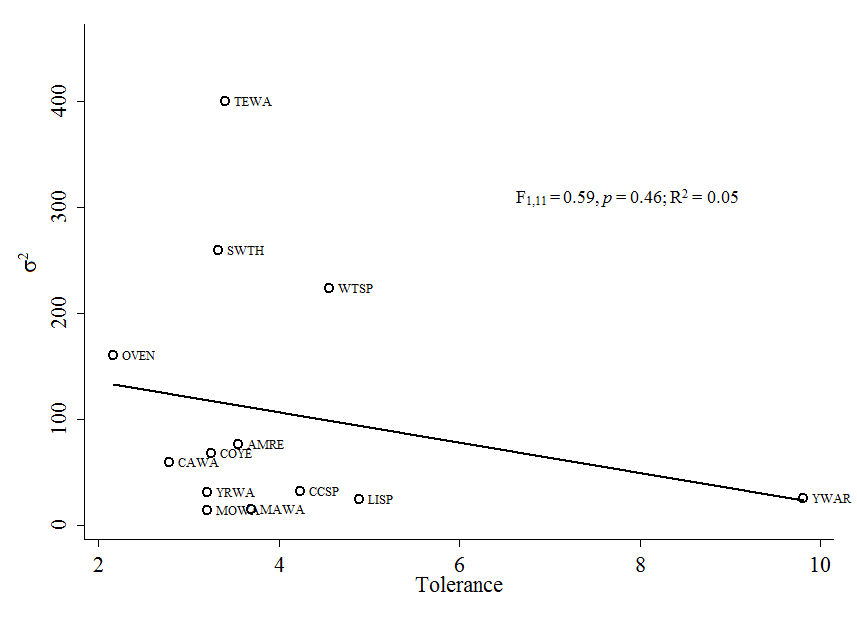

Supplement: S1 File — (DOCX) [file pone.0163957.s001.docx]
