# Supplementary material for: Within-Site Variation in Feather Stable Hydrogen Isotope (δ2Hf) Values of Boreal Songbirds: Implications for Assignment to Molt Origin
Source: PLoS One. 2016 Nov 2;11(11):e0163957. doi: 10.1371/journal.pone.0163957 (PMC5091831; doi:10.1371/journal.pone.0163957)
Supplement: S1 Table — (DOCX) [file pone.0163957.s002.docx]

**S1 Table. Candidate models ranked according to AICc weights (wAICc)**. K is the number of parameters estimated, AICc is the Akaike’s information criterion with correction for small sample size, ΔAICc is the difference between the AICc values of a given model and the best supported model, λ is the maximum likelihood estimate for the strength of the phylogenetic correlation.

| Model | K | AICc | ΔAICc | λ | wAICc |
| --- | --- | --- | --- | --- | --- |
| Het1 | 31 | 1422.2 | 0.0 | 0.00 | 0.74 |
| Het14 | 28 | 1425.2 | 3.0 | 0.00 | 0.16 |
| Het15 | 30 | 1427.4 | 5.2 | 0.00 | 0.06 |
| Het4 | 24 | 1428.2 | 6.0 | 0.29 | 0.04 |
| Het13 | 29 | 1435.5 | 13.2 | 0.06 | <0.01 |
| Het0 | 17 | 1445.0 | 22.8 | 0.84 | <0.01 |
| Het3 | 18 | 1446.1 | 23.9 | 0.83 | <0.01 |
| Het6 | 20 | 1446.7 | 24.5 | 0.84 | <0.01 |
| Het9 | 18 | 1446.9 | 24.7 | 0.83 | <0.01 |
| Het10 | 19 | 1447.1 | 24.9 | 0.84 | <0.01 |
| Het12 | 21 | 1447.2 | 25.0 | 0.71 | <0.01 |
| Het7 | 18 | 1447.3 | 25.1 | 0.83 | <0.01 |
| Het11 | 18 | 1447.4 | 25.2 | 0.83 | <0.01 |
| Het8 | 18 | 1447.5 | 25.3 | 0.83 | <0.01 |
| Het2 | 19 | 1448.9 | 26.7 | 0.81 | <0.01 |
| Het5 | 23 | 1453.5 | 31.3 | 0.82 | <0.01 |
| Hom1 | 17 | 1506.6 | 84.4 | 0.00 | <0.01 |
| Hom15 | 16 | 1509.1 | 86.9 | 0.00 | <0.01 |
| Hom14 | 14 | 1510.9 | 88.7 | 0.00 | <0.01 |
| Hom12 | 7 | 1523.5 | 101.3 | 0.57 | <0.01 |
| Hom9 | 4 | 1525.6 | 103.4 | 0.74 | <0.01 |
| Hom10 | 5 | 1527.5 | 105.3 | 0.74 | <0.01 |
| Hom0 | 3 | 1527.5 | 105.3 | 0.77 | <0.01 |
| Hom2 | 5 | 1528.2 | 105.9 | 0.72 | <0.01 |
| Hom13 | 15 | 1528.9 | 106.7 | 0.32 | <0.01 |
| Hom11 | 18 | 1529.1 | 106.9 | 0.77 | <0.01 |
| Hom7 | 4 | 1529.2 | 106.9 | 0.77 | <0.01 |
| Hom3 | 4 | 1529.6 | 107.4 | 0.78 | <0.01 |
| Hom8 | 4 | 1529.9 | 107.7 | 0.77 | <0.01 |
| Hom4 | 10 | 1531.7 | 109.5 | 0.58 | <0.01 |
| Hom6 | 6 | 1532.5 | 110.3 | 0.78 | <0.01 |
| Hom5 | 9 | 1534.7 | 112.5 | 0.79 | <0.01 |
