# Supplementary material for: Within-Site Variation in Feather Stable Hydrogen Isotope (δ2Hf) Values of Boreal Songbirds: Implications for Assignment to Molt Origin
Source: PLoS One. 2016 Nov 2;11(11):e0163957. doi: 10.1371/journal.pone.0163957 (PMC5091831; doi:10.1371/journal.pone.0163957)
Supplement: S2 Table — (DOCX) [file pone.0163957.s003.docx]

**S2 Table.** **Parameter estimates from the top-ranked model used to explain variation in δ^2^H_f_ among 192 adult migratory songbirds (Het1)**. This is a model with a fixed effect for species. Coefficients (β_j_) are provided for each species (AMRE being the reference category) along with an estimate of the lambda (λ) parameter to model the effect of phylogeny and species-specific variance estimates (σ^2^_j_).

| Parameter | Estimate | Lower 95% CI | Upper 95% CI |
| --- | --- | --- | --- |
| Intercept | -153.505 | 158.2525 | 148.7566 |
| β _CAWA_ | 9.4397 | 3.2575 | 15.6218 |
| β _CCSP_ | 8.9174 | 3.3499 | 14.4849 |
| β _COYE_ | 3.463 | -3.6477 | 10.5737 |
| β _LISP_ | 8.4531 | 3.0006 | 13.9056 |
| β _MAWA_ | 7.4582 | 2.2247 | 12.6917 |
| β _MOWA_ | 12.1833 | 7.0734 | 17.2933 |
| β _OVEN_ | 12.6364 | 3.6784 | 21.5943 |
| β _SAVS_ | -8.1406 | -13.9219 | -2.3593 |
| β _SOSP_ | 22.3365 | 9.1451 | 35.5279 |
| β _SWTH_ | 26.412 | 16.7154 | 36.1086 |
| β _TEWA_ | 22.4835 | 10.9796 | 33.9874 |
| β _WTSP_ | 14.9869 | 5.4405 | 24.5333 |
| β _YWAR_ | -1.437 | -6.9015 | 4.0274 |
| β _YRWA_ | 10.0668 | 4.436 | 15.6977 |
| λ | 0.0023 | -0.0123 | 0.0168 |
| σ^2^_AMRE_ | 76.5759 | 20.7972 | 132.3545 |
| σ^2^_CAWA_ | 59.3882 | 14.9211 | 103.8553 |
| σ^2^_CCSP_ | 31.6251 | 8.2407 | 55.0095 |
| σ^2^_COYE_ | 67.3951 | 5.5732 | 129.217 |
| σ^2^_LISP_ | 24.6955 | 4.8899 | 44.5011 |
| σ^2^_MAWA_ | 14.9865 | 1.5039 | 28.4691 |
| σ^2^_MOWA_ | 13.6193 | 3.0642 | 24.1744 |
| σ^2^_OVEN_ | 160.3174 | 20.0419 | 300.5929 |
| σ^2^_SAVS_ | 39.2899 | 10.1638 | 68.416 |
| σ^2^_SOSP_ | 460.7414 | 74.8595 | 846.6232 |
| σ^2^_SWTH_ | 259.4201 | 66.3039 | 452.5362 |
| σ^2^_TEWA_ | 399.5889 | 93.142 | 706.0358 |
| σ^2^_WTSP_ | 223.8819 | 52.3819 | 395.3819 |
| σ^2^_YWAR_ | 25.3968 | 4.7018 | 46.0918 |
| σ^2^_YRWA_ | 30.802 | 6.7798 | 54.8243 |
